# Supplementary material for: Cardinal v3 - a versatile open source software for mass spectrometry imaging analysis
Source: bioRxiv. 2023 Feb 21:2023.02.20.529280. Preprint. [Version 1] doi: 10.1101/2023.02.20.529280 (PMC9980127; doi:10.1101/2023.02.20.529280)
Supplement: Supplement 2 [file NIHPP2023.02.20.529280v1-supplement-2.pdf]

bioRxiv preprint doi: https://doi.org/10.1101/2023.02.20.529280; this version posted February 21, 2023. The copyright holder for this preprint (which was not certified by peer review) is the author/funder, who has granted bioRxiv a license to display the preprint in perpetuity. It is made available under aCC-BY 4.0 International license.

Supplementary Figures

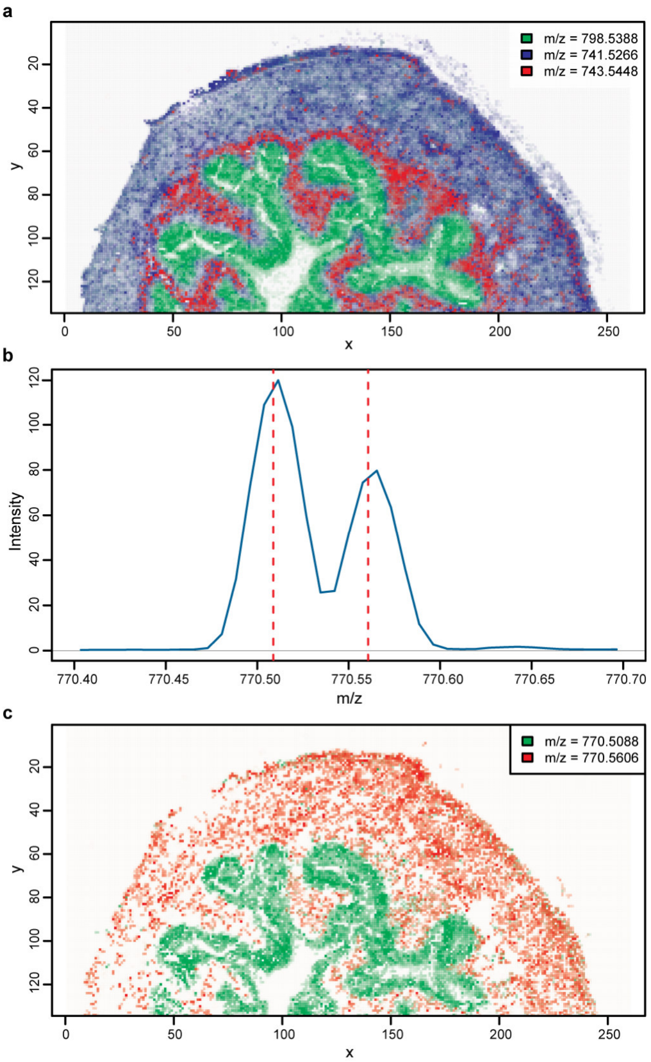

Supplementary Figure S1 - Cardinal 2 supports visualization and preprocessing of high mass resolution data

a, Reproducing the overlay ion image of three m/z features with different spatial abundances (Figure 1A in original publication). **b**, Zoomed in average mass spectra show two peaks that are only 0.05 m/z apart (Figure 2C in original publication) and could be detected as separate peaks. The red dotted lines indicate that both peaks will be picked separately during peak detection. **c**, Visualizing the spatial distribution of both peaks shows that they are present in different tissue regions (Figure 2B in original publication). The peaks in the original publication were likely mislabeled as suggested by Fig. S5 of the same publication and the inverse image we obtained here.

bioRxiv preprint doi: https://doi.org/10.1101/2023.02.20.529280; this version posted February 21, 2023. The copyright holder for this preprint (which was not certified by peer review) is the author/funder, who has granted bioRxiv a license to display the preprint in perpetuity. It is made available under aCC-BY 4.0 International license.

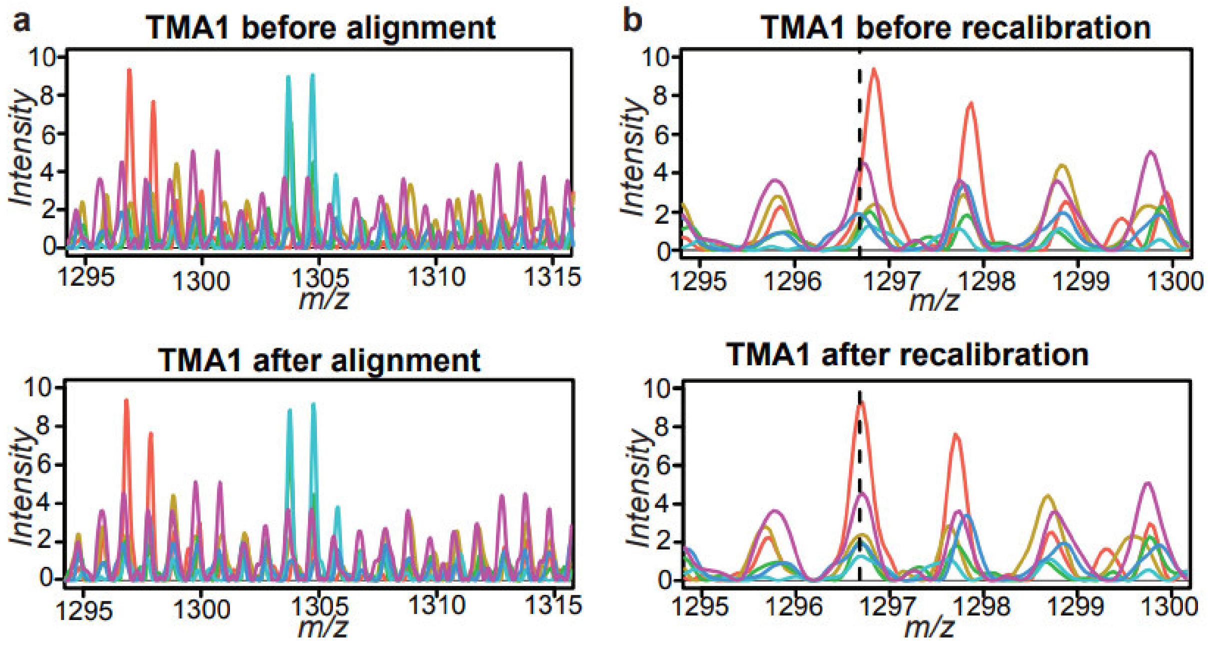

Supplementary Figure S2 - Cardinal 2 enables more accurate m/z values in the multiple replicates classification dataset

a, Zoomed in mass spectra for six random spectra of the first dataset show m/z shifts between the spectra before m/z alignment. Applying Cardinal's new mass alignment step increases the alignment of the peaks substantially.

b, Zoomed in mass spectra for six random spectra of the first dataset before and after mass re-calibration show how Cardinal's new mass re-calibration method shifts the monoisotopic angiotensin peak towards its theoretical m/z position (m/z 1296.7, dashed vertical line).

Supplementary information

Supplementary table 1: Cardinal documentation, training, support
